# Fixation strategies for periprosthetic femur fractures around knee replacements: a comprehensive single-center analysis of 102 consecutive cases

**DOI:** 10.1007/s00068-026-03252-y

**Published:** 2026-06-30

**Authors:** Josep Nomdedéu, Diego González-Morgado, Joan Minguell-Monyart, Nayana Joshi-Jubert, Jordi Teixidor-Serra, Jordi Tomàs-Hernández, José Vicente Andrés-Peiró

**Affiliations:** 1 Department of Surgery, Universitat Autònoma de Barcelona, Passeig Vall d’Hebron, 119-129, Barcelona, 08035 Spain; 2 Department of Orthopaedic Surgery, Hospital Universitari Vall d’Hebron, Barcelona, Spain; 3 Reconstructive Surgery of the Locomotor System Research Group, Vall d’Hebron Institut de Recerca (VHIR), Barcelona, Spain

**Keywords:** Periprosthetic knee fracture, Femoral fracture, Internal fixation, Fracture fixation, Treatment outcome, Postoperative complications, Healthcare costs

## Abstract

**Purpose:**

To evaluate 1 year mortality, complication rates, and functional outcomes after internal fixation of femoral periprosthetic knee fractures following total knee arthroplasty, and to identify predictors of adverse outcomes and increased healthcare resource utilization.

**Methods:**

This retrospective cohort study included 102 consecutive patients with femoral periprosthetic knee fractures classified as UCPF VB3 and VC3 treated with internal fixation at a single tertiary center between 2010 and 2023. Interprosthetic fractures and combined fixation techniques were excluded. Primary outcomes were 1 year mortality and postoperative complications. Secondary outcomes included length of stay, discharge destination, and ambulatory status at follow up.

**Results:**

Patients were markedly frail, with a mean age of 82.4 ± 10.8 years and mean Charlson Comorbidity Index of 5.6. One year mortality was 16.7% and was associated with older age, higher ASA class, higher Charlson index, impaired pre fracture ambulation, and fracture related infection. Overall complication rate was 29.4%, including 15.7% infections and 13.7% mechanical failures. Open reduction was more frequent in plate fixation and resulted in longer operative times, without increasing complication rates. Median hospital stay was 18 days, 66.3% required discharge to nursing facilities, and only 7.8% regained independent ambulation. Outcomes were worse in patients who developed complications.

**Conclusion:**

Outcomes after internal fixation of femoral periprosthetic knee fractures are driven primarily by patient frailty rather than fixation technique. Functional recovery is limited and resource utilization is substantial, underscoring the need for improved risk stratification and perioperative care pathways.

## Introduction

The number of total knee arthroplasties (TKA) being performed is steadily increasing, driven by the longer life expectancy of the population and the dramatic rise in certain risk factors for osteoarthritis. In the United States, the number of TKAs is projected to reach 3.48 million by 2030 [1]. Although total knee arthroplasty (TKA) is a highly successful treatment for patients with osteoarthritis, it is not without risks. Among its complications, periprosthetic knee fractures (PKFs) are particularly concerning. The incidence of femoral PKFs ranges from 0.3% to 5.5% following primary TKA and may rise to as high as 30% in revision procedures [1–4]. Traditionally, fixation of femoral PKFs has been indicated for simple fracture patterns occurring around stable prostheses and with sufficient distal bone stock [5–9]. Distal femoral replacement (DFR), although a valid alternative, presents disadvantages when compared to fixation, such as longer operative times and a more invasive surgical approach [10, 11]. While DFR has traditionally been reserved for the most complex cases, recent advances in osteosynthesis techniques have increasingly enabled internal fixation in a broader range of fracture patterns. These include the use of periprosthetic-specific locking plates, dual plating systems, and combined nail and plate constructs, which have demonstrated outcomes comparable to those of DFR [12–15]. Given the high morbidity, mortality, and economic burden associated with periprosthetic knee fractures, these innovations are crucial for preserving patient quality of life and healthcare sustainability [1, 4, 16, 17]. However, a better understanding of the factors that influence fixation techniques remains essential.

The primary aim of this study was to assess mortality rates and complication profiles associated with fixation procedures for femoral periprosthetic knee fractures, and to identify the factors influencing each of these outcomes. We hypothesize that our findings will align with previously reported data and that patients with a more compromised baseline status or complex fractures requiring more aggressive surgical strategies will have a higher risk of adverse events. A secondary analysis will examine disability-related outcomes and healthcare resource utilization.

## Materials and methods

We obtained approval from our Institutional Review Board prior to initiating the present study (code PR(AT)296/2022). This is a retrospective cohort study (level of evidence III) on patients treated at a single level III public and university hospital between February 2010 and March 2023. We included all skeletally mature patients sustaining femoral fractures around knee replacements (Unified Classification System for Periprosthetic Fractures “UCPF” types VB3 and VC3) [6] treated through fracture fixation excluding fractures dividing the bone between two implants (interprosthetic and interimplant fractures). To better compare intramedullary and extramedullary fixation, patients with augmentation or combined implants were excluded. All procedures were performed by a team of surgeons specializing exclusively in fracture fixation and with extensive experience in managing periprosthetic fractures.

Patient data were retrospectively collected for the first year of postoperative follow-up. The data included baseline characteristics of the patients and their arthroplasties, fracture details, treatment modalities, and outcomes. Fractures were classified using the UCPF, Lewis-Rorabeck, and Su classifications [6–8]. The primary outcomes were mortality and the occurrence of postoperative local complications, categorized as either infectious or mechanical. Fracture-related infection (FRI) was defined based on clinical, radiological, and laboratory criteria [18]. Mechanical complications were defined as the occurrence of nonunion, malunion, or implant-related issues requiring revision surgery. Secondary outcomes included the length of hospitalization, the need for convalescence in a nursing care facility, and deterioration in ambulatory capacity. Ambulatory capacity was assessed before the fracture and at final follow up. Four ordinal categories were used: independent ambulation, one aid, two aids or walker, and non-ambulatory. Deterioration was defined as a decline of at least one category. Cases with missing data at either time point were excluded from the functional analysis.

Statistical analyses were conducted using Stata/IC 14.2 (StataCorp LLC, USA). Continuous variables were described using means and standard deviations or medians and interquartile ranges, depending on their distribution. Categorical variables were presented as percentages. Quantitative variable comparisons were conducted using Student’s t-test or the Wilcoxon-Mann-Whitney test, as appropriate, while ANOVA was used for comparisons involving three or more groups. Associations between categorical variables were analyzed using chi-squared or Fisher’s exact tests. Correlations between continuous variables were assessed using Pearson’s correlation coefficient (r) and corresponding *p*-values. Survival analysis was performed using the Kaplan-Meier method. Statistical significance was defined as *p* < 0.05.

## Results

### Sample composition

**Fig. 1:**
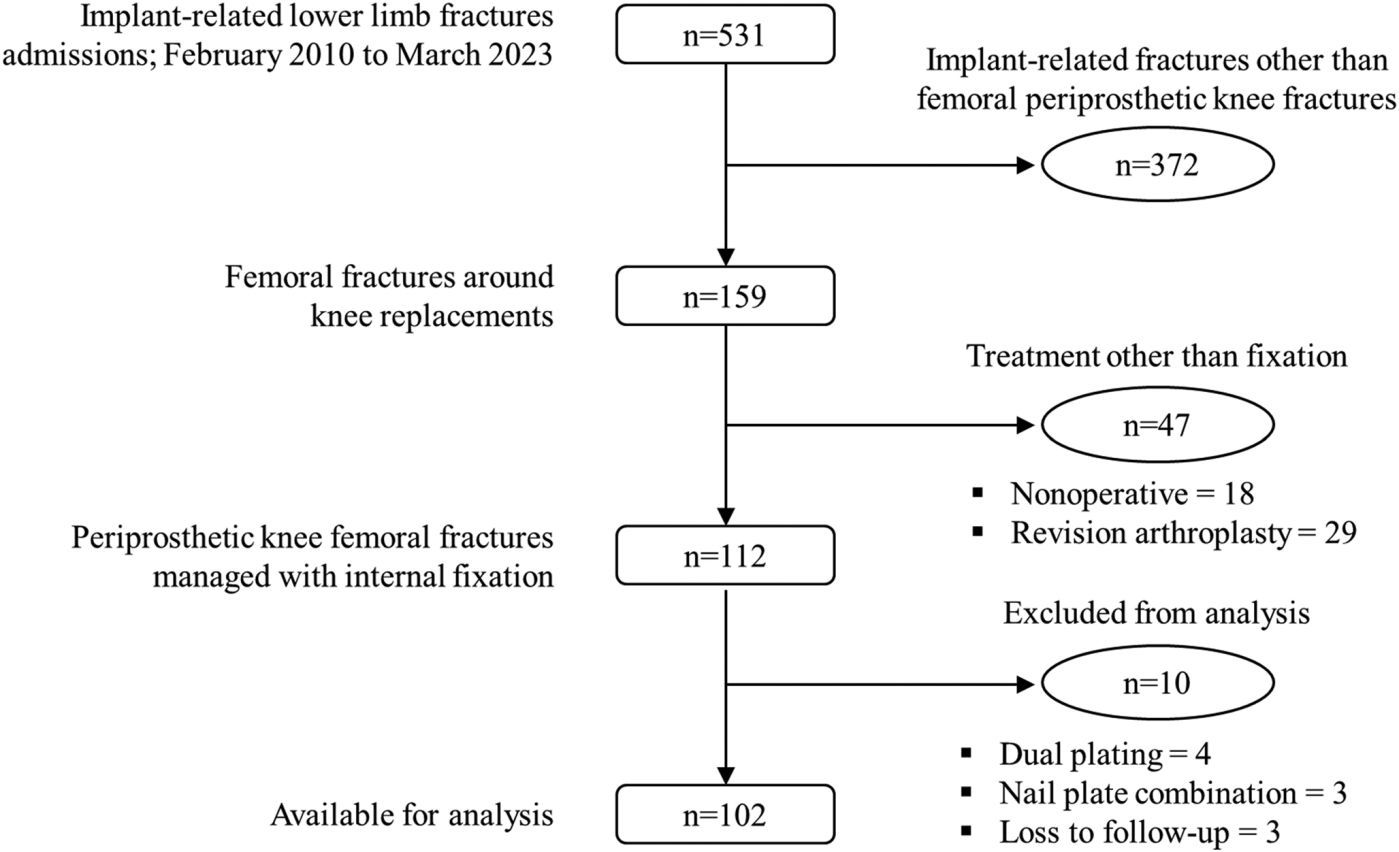
Flow diagram of the patient selection process

**Fig. 2:**
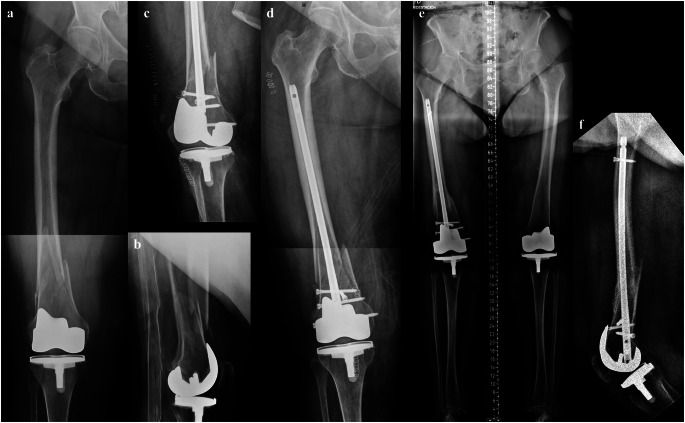
Treatment of a Su type I and Lewis–Rorabeck II fracture with a retrograde intramedullary nail. Preoperative images (**a, b**) show a comminuted postisthmic fracture proximal to a primary total knee arthroplasty with a cruciate retaining femoral component. Postoperative images (**c, d**) demonstrate satisfactory stabilization, supplemented with a distal poller screw to optimize alignment. At one year (**e, f**), coronal alignment is anatomical. A mild recurvatum deformity is observed on the lateral view, attributable to a posterior nail entry point, without clinical relevance. Nail designs with increased Herzog curvature may help minimize this effect

**Fig. 3:**
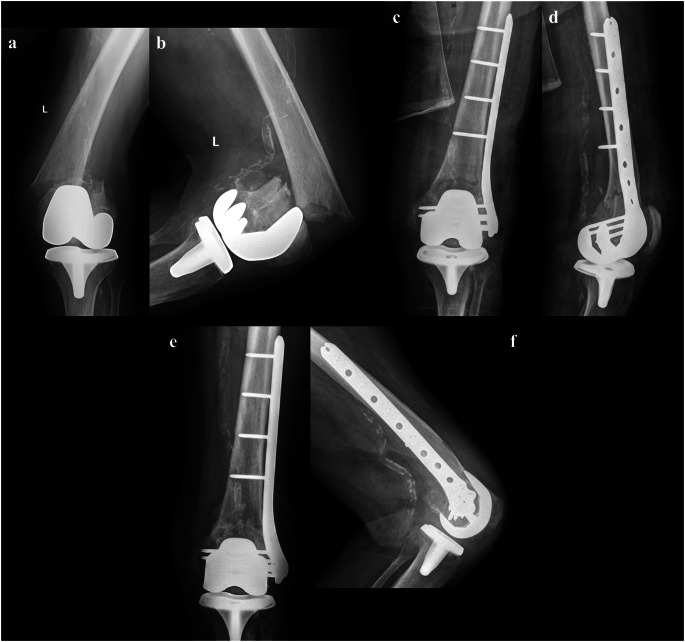
Treatment of a Su type II and Lewis–Rorabeck II fracture using a lateral distal femur anatomical plate. Preoperative images (**a, b**) show a transverse fracture with severe anterior displacement, located immediately proximal to the femoral component shield. Postoperative images (**c, d**) demonstrate satisfactory stabilization with a lateral bridge plate. The marked displacement required an open reduction. At one year (**e, f**), both coronal and sagittal alignment are anatomical

We identified 102 cases available for analysis (Fig. 1), comprising 95 women and 7 men, with a mean age of 82.4 ± 10.8 years. The average score on the age-adjusted Charlson Comorbidity Index (aa-CCI) was 5.6 ± 1.8, reflecting severe comorbidity [19]. Furthermore, 97.6% of the patients had moderate to severe comorbidities as classified by the American Society of Anesthesiologists physical status classification system (ASA ≥ II) [20]. At the time of fracture presentation, 60.8% of patients exhibited impaired ambulation, and 16.7% resided in nursing facilities, findings that were associated (*p* = 0.01). A total of 86.3% of arthroplasties were cemented, and 14.7% involved stemmed implants. Records on prior arthroplasties were available for 59.8% of cases, with an average interval of 8.2 ± 5.2 years between replacement and fracture. Among these, 9.8% exhibited signs of arthroplasty compromise, including pain, a history of infection, or other complications. The most frequent fracture patterns were Lewis-Rorabeck II (85.3%), Su I and II (40.2% and 54.9%, respectively) and UCPF VB3-1 (64.7%). In cemented prostheses, fractures were predominantly of the Rorabeck II type (90.4%), whereas in uncemented prostheses, the distribution was more balanced between type I (42.9%) and type II (50%) (*p* < 0.01). Intramedullary nailing was performed in 22.5% of cases, and plate fixation was used in 75.5%, frequently supplemented with cerclage wiring, which was used in 41.2% of cases. Open reduction was performed in 87.3% of cases, of which 60.7% used a midline knee approach, and 39.3% used a lateral subvastus approach to the fracture focus. The need for open reduction was associated with a higher classification in the Lewis-Rorabeck system (80% vs. 73.2% vs. 100% for types I, II, and III, respectively; *p* < 0.01) and was more common with plate compared to intramedullary fixation (93.5% vs. 68%; *p* < 0.01). The mean operative time was 115.5 ± 50.8 minutes, with a tourniquet used in 9.8% of cases. Longer operative times were observed in cases requiring open reduction (124.3 vs. 89.8 minutes; *p* < 0.01) and in higher categories of the Lewis-Rorabeck classification (104.8 vs. 118.9 vs. 177.6 minutes for types I, II, and III, respectively; *p* = 0.03). Table 1 provides a comprehensive description of independent variables. Figures 2 and 3 provide representative examples of the fixation strategies most frequently used in our series.

**Table 1:** Baseline characteristics of patients, their injuries, and applied treatments

	Independent variable	Results^1^
Patients	Age (years)	82.4 ± 10.8
	Sex (female/male)	93.4%/6.9%
	aa-CCI^2^	5.6 ± 1.8
	ASA^3^	
	▪ I	3 (2.9)
	▪ II	22 (21.6)
	▪ III	71 (69.6)
	▪ IV	6 (5.9)
	Impaired ambulation^4^	
	Nursing facility^5^	
Injuries	Stemmed femoral implant	15 (14.7)
	Cemented arthroplasty	88 (86.3)
	Lewis-Rorabeck^6^	
	▪ I	10 (9.8)
	▪ II	87 (85.3)
	▪ III	5 (4.9)
	Su^7^	
	▪ I	41 (40.2)
	▪ II	56 (54.9)
	▪ III	5 (4.9)
	UCPF^7^	
	▪ VB3-1	66 (64.7)
	▪ VB3-2	2 (2)
	▪ VB3-3	3 (2.9)
	▪ VC3	20 (19.6)
	▪ VD3	11 (10.8)
Treatments	Time of surgery (minutes)	120 ± 50.8
	Tourniquet	10 (9.8)
	Open reduction	89 (87.3)
	Surgical approach	
	▪ Closed/mini-open	13 (12.8)
	▪ Midline lateral parapatellar	45 (44.1)
	▪ Midline medial parapatellar	9 (8.8)
	▪ Lateral subvastus	35 (34.3)
	Fixation	
	▪ Intramedullary (nailing)	25 (24.5)
	▪ Extramedullary (plating)	77 (75.5)

^1^Categorical variables are presented as n (%); continuous variables as mean ± standard deviation (SD). ^2^Age-adjusted Charlson Comorbidity Index. ^3^American Society of Anesthesiologists (ASA) Physical Status Classification System. ^4^Use of one walking aid, two walkingaids, a walker, or non-ambulatory status. ^5^Nursing facility resident. ^6^Lewis and Rorabeck classification of femoral periprosthetic fractures. ^7^Su classificationof femoral periprosthetic fractures. ^8^Unified Classification System (UCS) for Periprosthetic Fractures

### Mortality and complications

**Fig. 4:**
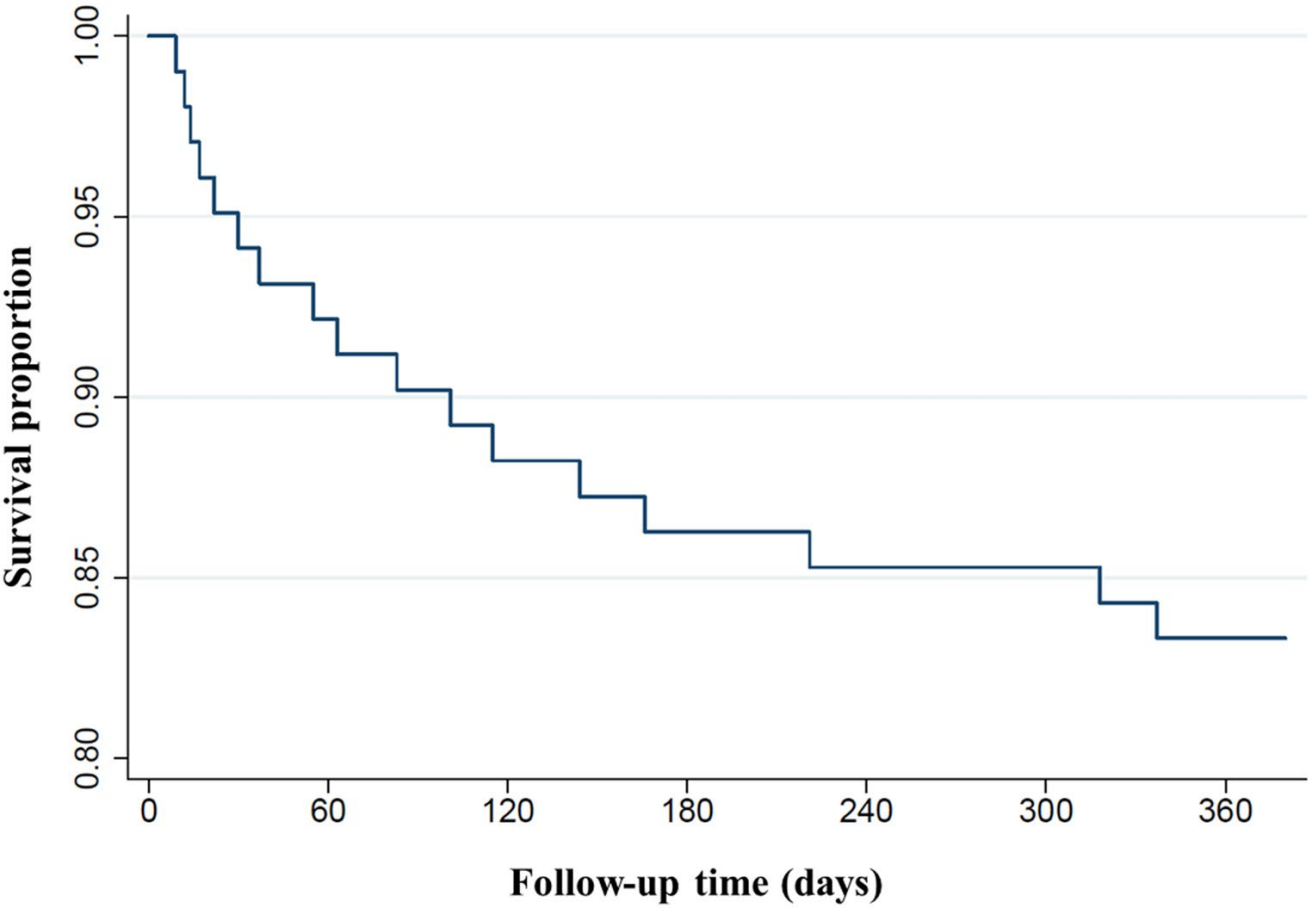
Kaplan–Meier survival curve for first postoperative year mortality. The survival curve demonstrates that the majority of deaths were concentrated within the first three months after osteosynthesis

Seventeen of 102 patients (16.7%) died within the first-year post-fixation, including five during hospitalization (Fig. 4). Mortality was associated with older age (88.3 vs. 81.2 years; *p* < 0.01), higher ASA (3.1 vs. 2.7; *p* = 0.02) and aa-CCI scores (2.7 vs. 1.8; *p* = 0.02), impaired preoperative ambulation (22.6% vs. 7.5%; *p* = 0.04), and fracture-related infection (37.5% vs. 12.8%; *p* = 0.03). Overall, 29.4% of patients experienced local postoperative complications, with 15.7% developing fracture-related infections and 13.7% experiencing mechanical complications (Table 2). Mechanical complications occurred only in Lewis-Rorabeck type II fractures and Su type I and II fractures. In type I fractures, plate fixation resulted in twice as many mechanical failures compared to nailing. However, none of these differences reached statistical significance. No cases of capsular suture failure or extensor mechanism necrosis were observed, even with iterative midline approaches and external parapatellar access in 44.1% of cases. Patients living at home before the fracture had more complications (34.1% vs. 5.9% in care facilities; *p* = 0.02). No other significant associations were found between patient characteristics, injury patterns, or treatments and complications.

**Table 2:** Summary of complications and their management

Complication	Diagnosis	Management
Fracture-related infection = 16	Postoperative SSI^1^ = 12	DAIR^2^ = 11
		Nonoperative (sepsis-related death) = 1
	Infected nonunion = 3	Distal femoral replacement = 3
	Late hematogenous infection = 1	Implant removal (consolidated fracture) = 1
Mechanical complications = 14	Aseptic nonunion = 7	Distal femoral replacement = 2 Revision of fixation = 5 ▪ 4 plate to nail ▪ 1 plate to NPC^3^
	Failure of fixation = 5	Distal femoral replacement = 3 Revision of fixation = 5 ▪ 1 plate to nail ▪ 1 plate to NPC^3^
	Inter-implant fracture = 1	Revision of fixation (nail to plate) = 1
	Painful total knee replacement = 1	Locking screw removal and patella prosthetization = 1

^1^SSI: surgical site infection

^2^DAIR: debridement and implant retention

^3^NPC: nail-plate combination

### Resource consumption and disability

The median hospital stay was 18 days (IQR = 50), with a median delay from admission to fracture fixation of 6 days (IQR = 4). We observed a weak correlation between prolonged hospitalization and the aa-CCI score (*r* = 0.21; *p* = 0.04) and a moderate correlation between hospitalization duration and the need for transfusion (*r* = 0.52; *p* < 0.01). Hospital stays were also longer for patients discharged to a nursing facility when they had not previously lived in one, compared to those discharged home (26.3 vs. 17.3 days, respectively; *p* = 0.03). No clinically relevant associations were identified between prolonged delay to fixation and other factors, like the use of anticoagulant drugs.

Excluding patients who were already living in nursing homes prior to surgery and those who died during hospitalization, 66.3% of patients required convalescence in a nursing facility after fracture fixation. This outcome was significantly associated with female sex (69.3% vs. 20%; *p* = 0.04) and worse baseline conditions, including older age (84.7 vs. 79 years; *p* < 0.01), higher ASA scores (2.9 vs. 2.3; *p* < 0.01), and the use of antiplatelet or anticoagulant medications (83.3% vs. 58.9%; *p* = 0.03).

At the end of the follow-up period, 92.2% of patients experienced ambulatory limitations compared to 60.8% preoperatively, reflecting a 31.4% decline, with 36.3% becoming non-ambulatory. This deterioration was significantly more pronounced in patients who recovered in care facilities (98.1% vs. 74.1%; *p* < 0.01) and those who developed complications (50% vs. 23.6% in patients without complications; *p* < 0.01). The time from surgery to ambulation initiation averaged 9.3 ± 6.8 weeks, being longer in patients treated with plates compared to nails (10.9 vs. 5.7 weeks; *p* < 0.01) and in those with postoperative complications (11.6 vs. 7.5 weeks; *p* = 0.02).

## Discussion

In our cohort of 102 periprosthetic knee fractures treated with internal fixation, frail and elderly patients predominated (mean age 82.4 years), as did open reduction (87.3%) and plate fixation (75.5%). Most cemented TKAs were associated with LR II fractures, while LR I were evenly distributed between cemented and uncemented. Although cementation has been linked to a lower incidence of PKF, it does not appear to influence fracture classification, and its impact on fixation outcomes remains unclear [3]. One-year mortality was 16.7%, with higher rates observed in older, comorbid and functionally impaired patients, and those who developed fracture-related infections. Postoperative complications occurred in 29.4% of cases, including 15.7% infections and 13.7% mechanical failures. Hospitalizations were prolonged (median 18 days), and 66.3% of patients required discharge to a care facility. Ambulatory limitations persisted in 92.2% of patients at final follow-up.

In our series, plate fixation was the most employed method. Retrograde nailing was limited to Su type I fractures when compatible with the TKA design, whereas types II and III were primarily managed with plating. When possible, nailing was preferred due to its biological advantages and superior soft tissue preservation [2, 5, 21–26]. Very distal fractures or cases with compromised distal bone stock often required plate fixation to achieve anatomical reduction, which correlated with increased operative time [4, 12, 15, 21, 22, 27]. Open reduction was more frequent with plates and associated with longer surgical durations, as previously reported [1]; all LR III cases required it, extending operative time by nearly one hour. Because nailing was restricted to Su type I fractures and plating was used for more distal or complex patterns, between-technique differences should be interpreted as descriptive associations conditioned on fracture pattern and baseline risk. Current evidence suggests that both nailing and plating provide equivalent outcomes in terms of mortality and complication rates [14, 21–31], which was also observed in our cohort. When using plates, angular stable constructs have been associated with reduced rates of mechanical failure [1, 2, 4, 9, 13, 32, 33]. Recent advances in implant design and surgical technique may broaden indications and improve results. In osteoporotic bone, cement-augmented nailing has shown promising outcomes [14]. Modern low-profile nails facilitate insertion through prosthetic components, although complications such as screw back-out persist [34]. In cases with medial comminution or limited bone stock, strategies such as medial or double plating have been described in the literature [15]; such constructs were not used in our cohort. Combined constructs using nailing and plating may offer enhanced stability, albeit with increased operative time and healthcare costs [5, 12]. The lateral parapatellar approach was used in 44.1% of our cases. Repeated lateral tenotomies or extensive releases have been linked to capsular suture failure and patellar tendon necrosis [10]. In our series, no cases of capsular failure or extensor mechanism necrosis were observed, supporting the safety of midline approaches in this context.

The one-year mortality rate was 16.7%, higher in older patients and those with comorbidities (ASA, Charlson), impaired ambulation, or compromised arthroplasty. Although these are nonmodifiable factors, they may help identify particularly frail patients at higher risk [4, 35, 36]. In our cohort, complications occurred in 29.4% of patients, with 13.7% being mechanical and 15.7% infectious, within the reported range of 16–42% [21, 25, 28, 36]. The slightly elevated infection rate may reflect the cohort’s frailty. Women represented 93% of patients, the median age exceeded typical reports by six years (70–80 years), and the mean Charlson Index was 5.6, higher than the commonly reported range of 4.4 to 4.9 [4, 5, 21, 25, 28, 29, 35, 36]. A notable finding was the significantly higher complication rate in patients living at home prior to the fracture compared to those from care facilities (34.1% vs. 5.9%, *p* = 0.02). One possible explanation is that institutionalized patients, often discharged to nursing homes, experience earlier mortality, potentially reducing the time window for complications to develop or be detected. Previous studies have reported a 14-fold increase in mortality among patients discharged to such facilities, supporting this hypothesis [37]. The high rate of open reductions in our series may have contributed to the elevated infection rate, as this approach increases exposure, contamination and operative time, all factors associated with a higher risk of postoperative infection [38]. Although minimally invasive techniques may help preserve the biological environment, most authors emphasize the importance of anatomical reduction to prevent mechanical failure [5, 13, 15, 31]. This often requires open reduction, which may disrupt the biological environment and increase the risk of infection [13, 31, 39–42]. However, we found no significant association between open reduction and infection, and previous studies have not consistently reported its frequency or impact in PKF management.

PKFs severely affect functional status, especially ambulation. While previous studies report impaired walking in 70–79% of patients regardless of fixation method [1, 4, 25, 30, 43], fewer than 10% of our patients walked unaided at final follow-up. This may reflect the cohort’s advanced age, nearly 10 years older than in some series, as age strongly predicts functional recovery [44]. Outcomes were worse in those discharged to nursing homes, with over 30% showing deterioration and 36.4% losing ambulation entirely. Patients treated with intramedullary nails recovered ambulation faster, although modern locking plates also allow early weightbearing without increased complications [45, 46].

PKFs place a substantial burden on healthcare systems due to prolonged hospitalization, complex management, high complication and readmission rates, and frequent need for post-discharge care [1, 4, 16, 17]. One study found that 75% of patients did not return home and 25% of those discharged to nursing facilities were readmitted within 30 days, mostly for infection [16]. In our cohort, 66.3% were not discharged home, and older, frailer patients had hospital stays up to 10 days longer. With increasing PKF incidence and reliance on nursing facilities, this burden may become unsustainable. Early discharge strategies may help reduce complications and overall costs.

We acknowledge several limitations of our study, including its retrospective design, lack of randomization, and single-center setting, which may limit the generalizability of our findings. The non-random allocation prevents comparison between techniques, and the differences between groups preclude valid statistical adjustment. The ambulatory outcome was ascertained retrospectively rather than through a validated scale, which may introduce information bias. Additionally, data on prior arthroplasty were incomplete in a substantial proportion of cases. In many of these, prosthesis details were unavailable, limiting surgical planning and potentially affecting outcomes. In the future, standardized implant identification systems may facilitate preoperative planning and improve clinical decision-making. Furthermore, standardized patient-reported outcome measures to assess functional recovery were not available. Nevertheless, the inclusion of a large, consecutive cohort treated by an experienced surgical team, together with a comprehensive evaluation of clinical outcomes and healthcare resource utilization, provides robust and clinically relevant insights into the internal fixation of femoral periprosthetic knee fractures.

## Conclusion

In our cohort of femoral periprosthetic knee fractures treated with internal fixation, patient-related factors such as age, comorbidities, and baseline function were more strongly associated with mortality and complications than the fixation method itself. Open reduction was common and associated with longer operative times, although no increased risk of complications was demonstrated. Functional recovery was poor, with fewer than 10% of patients regaining independent ambulation. Resource utilization was substantial, with prolonged hospital stays and frequent discharge to nursing facilities.

## Data Availability

The data that support the findings of this study are available from the corresponding author, JVAP, upon reasonable request.
